# Surgical management of a giant venous aneurysm in an autogenous arteriovenous fistula with the vessel loop shoelace technique for wound closure: A case report

**DOI:** 10.1097/MD.0000000000028072

**Published:** 2021-12-03

**Authors:** Young Sun Yoo

**Affiliations:** Department of Surgery, College of Medicine, Chosun University, Gwangju, South Korea.

**Keywords:** arteriovenous fistula, basilic vein, case report, giant venous aneurysm, hemodialysis

## Abstract

**Rationale:**

Giant venous aneurysm (GVA) is a complication of chronic arteriovenous fistula (AVF). The risks of thrombosis, rupture, and massive hemorrhage increase with the increasing size of GVA; therefore, GVA requires treatment. However, the optimal timing and treatments are yet to be established.

**Patient concerns:**

A 51-year-old male patient who had been undergoing hemodialysis for 10 years using a left radio-cephalic AVF presented to the hospital with an enormous venous aneurysm.

**Diagnosis:**

Physical examination and ultrasound revealed a GVA in the AVF.

**Intervention:**

The aneurysm was resected, and autogenous AVF was concomitantly formed using the basilic vein. The large wound caused by the removed aneurysm defect was closed using the vessel loop shoelace technique.

**Outcomes:**

The wound healed completely, and the patient has been undergoing hemodialysis using the autogenous AVF created during the surgery for 15 months since then.

**Lessons:**

Surgical treatment should be considered for symptomatic GVA. Concomitant aneurysm resection and autogenous AVF formation using the basilic vein may be performed, and the resulting large wound can be closed with the vessel loop shoelace technique to facilitate healing.

## 1 Introduction

Venous aneurysm of a hemodialysis access fistula is a relatively common complication of prolonged use of an arteriovenous fistula (AVF). In general, a venous aneurysm in an AVF is defined as a 2 to 3 times increase in diameter compared to surrounding normal veins or venous expansion of 2 cm or larger, and the prognosis differs according to the site and shape of the expansion.^[1,2]^ Although there is no specific criterion for the size, some authors refer to enormous venous aneurysms as giant venous aneurysms (GVA).^[3–6]^

Generally, venous aneurysms are not problematic because they do not affect hemodialysis function. The indications for treatment include patient discomfort or cosmetic concerns, risk of bleeding, low flow issues due to outflow stenosis, and high flow issues such as steal syndrome or high output cardiac failure.^[7]^ Further, massive enlargement of venous aneurysms may cause thinning of the skin overlying the aneurysm, and ulceration or infection of the skin may cause bleeding, calling for immediate treatment. The 2019 update to the Kidney Disease Outcomes Quality Initiative Clinical Practice Guideline for Vascular Access recommends considering surgical treatment as the first line of treatment in cases of symptomatic, large, or rapidly expanding aneurysms.^[8]^

The surgical treatment for venous aneurysms involves aneurysm resection followed by the interposition of a prosthetic graft. However, resecting the aneurysm would sacrifice the entire AVF in cases of GVA, so another dialysis access must be created, which makes it difficult to decide on the optimal timing and method of surgery. In addition, the GVA itself is quite large, so removing the aneurysm leaves a large wound, and primary closure is often challenging owing to the tension in the wound caused by skin retraction.

Here, we report a case of GVA caused by long-term use of an AVF that was treated with concomitant aneurysm resection and creation of an autogenous AVF, followed by the closure of the resulting large wound using the vessel loop shoelace technique.

## 2 Case

**Figure 1:**
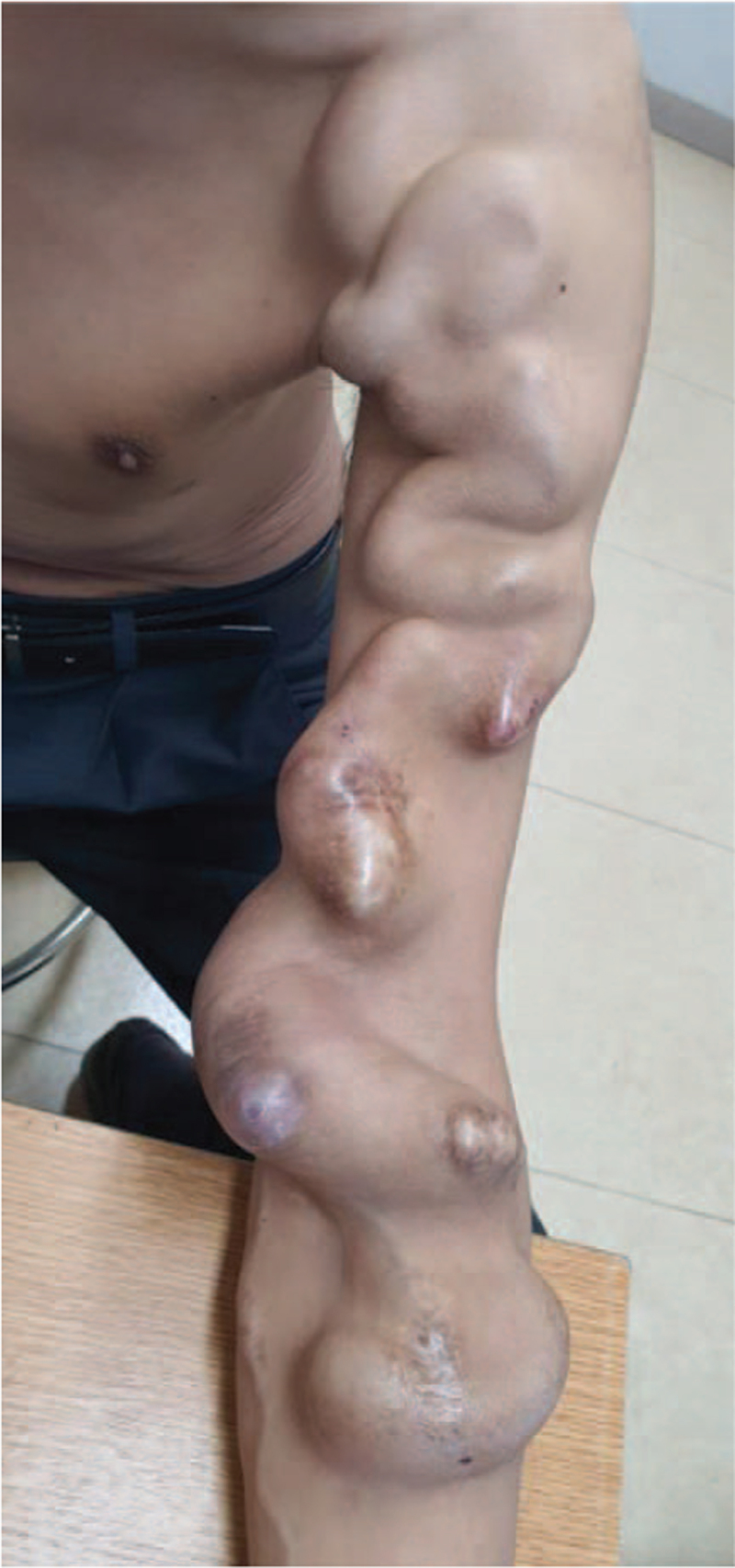
Extensive GVA spanning the forearm and upper arm. GVA = giant venous aneurysm.

A 51-year-old male patient had begun hemodialysis 15 years earlier owing to end-stage renal failure caused by an exacerbation of glomerulonephritis. A left radio-cephalic AVF had been created at the time for hemodialysis. Ten years previously, the patient experienced failure of the radio-cephalic AVF. At a different hospital, he underwent the creation of a left proximal radio-cephalic AVF by an anastomosis of the mid cephalic vein with the mid radial artery located 15 cm below the antecubital crease. Although aneurysmal changes in the AVF began several years ago, the patient continued to undergo hemodialysis without regular check-ups because there were no functional impairments in hemodialysis. Finally, he presented to our hospital when the vein was enormously dilated (Fig. 1).

The patient wanted removal of the venous aneurysm because of the heaviness of the arm and poor cosmetic appearance, but he wanted us to create a new access point using an autogenous vein instead of an artificial graft. Preoperative ultrasound confirmed an extensive aneurysmal change in the AVF with a maximum diameter of 4 cm. We checked for available autogenous veins and found that the basilic vein in the upper arm had a diameter of at least 3 mm with an adequate length; hence, we decided to perform a concomitant resection of the venous aneurysm and anastomosis of the basilic vein with the brachial artery to create an AVF. Preoperative echocardiography confirmed no deteriorating cardiac function. To secure dialysis access postoperatively, we inserted a temporary dialysis catheter into the right internal jugular vein, and a venogram taken at the time confirmed there was no central vein stenosis.

**Figure 2:**
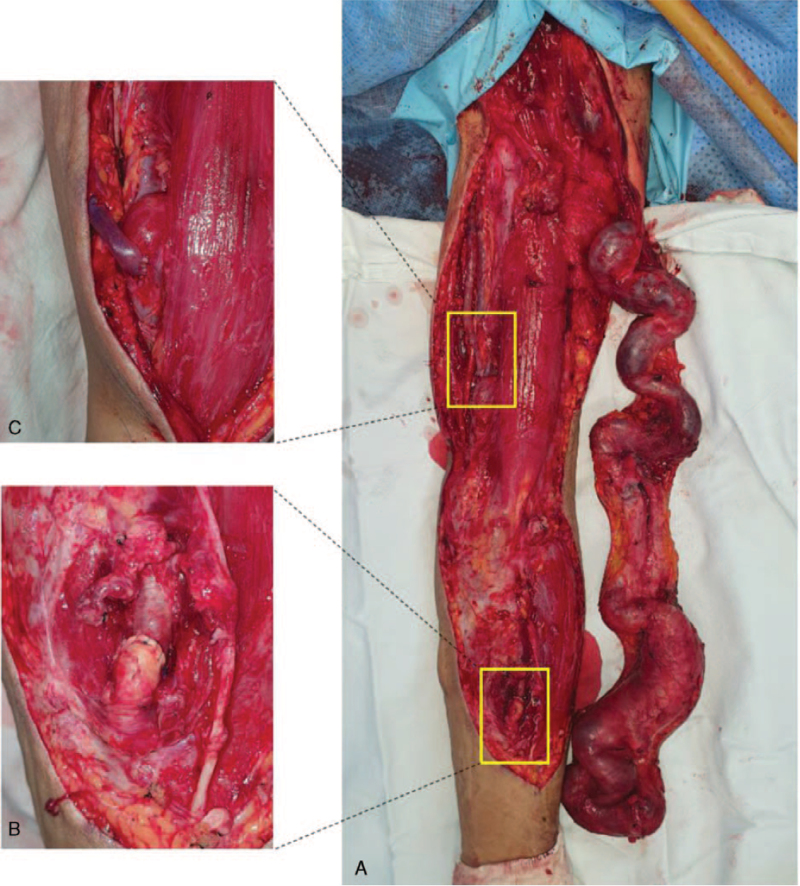
(A) The entire aneurysm in the AVF was dissected and skeletonized. (B) After removing the aneurysm, the proximal radial artery was connected via end-to-end anastomosis. (C) After dissection, the exposed upper arm basilic vein was anastomosed to the brachial artery to form an AVF. AVF = arteriovenous fistula.

Under general anesthesia, a longitudinal skin incision was made around the thin layer of the skin above the aneurysm and cannulation site to expose the entire aneurysm and the anastomosis between the radial artery and cephalic vein, and the dilated venous aneurysm of the forearm and upper arm were circumferentially and completely skeletonized (Fig. 2A). After clamping the proximal and distal segments of the radial artery, the entire venous aneurysm was removed, and arterial continuity was maintained through end-to-end anastomosis of the radial artery (Fig. 2B). The upper arm basilic vein was identified, dissected, and connected to the brachial artery via end-to-side anastomosis, and a thrill was palpated (Fig. 2C). We also confirmed that the radial artery pulse in the wrist was intact.

**Figure 3:**
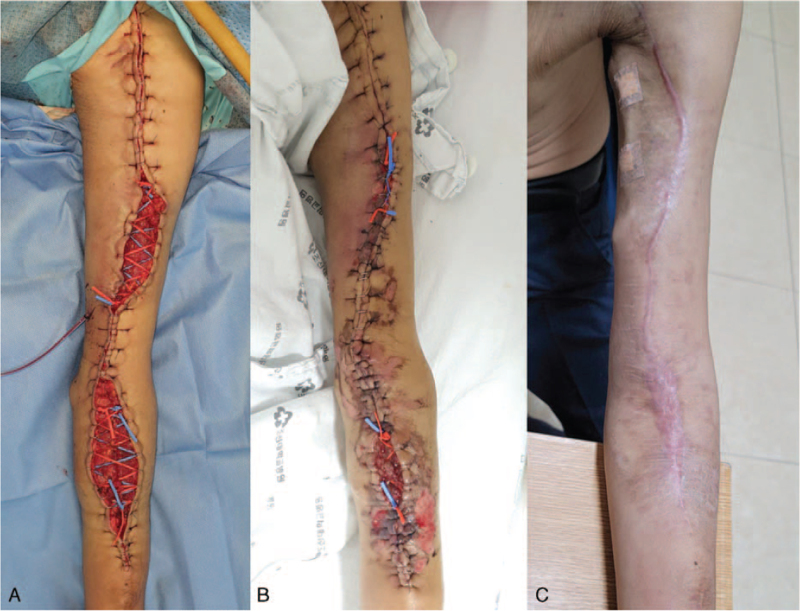
(A) The wound is closed using the vessel loop shoelace technique. (B) On POD 10, the wound was gradually closed, and the wound margin was almost completely closed. (C) On POD 30, the wound was completely healed, and the basilic vein was matured enough for hemodialysis. POD = postoperative day.

After meticulous hemostasis, we placed a suction drain in the wound, positioned the upper basilic vein in the subcutaneous layer, checked the thrill, and began wound closure. We approximated the skin margins and used 3-0 nylon for the closure but had difficulty because of the severe tension caused by skin retraction in the mid portion of the forearm and upper arm. Hence, we decided to apply the vessel loop shoelace technique for gradual traction of the wound edge. Staples were placed on the skin edge where skin traction could not be performed, and a vessel loop was passed through the staples and pulled as much as possible to fix the skin, as in tying shoelaces (Fig. 3A). The total length of surgery was 4 hours and 30 minutes, and the estimated blood loss was 300 cc. No blood transfusions were performed during the surgery.

During routine postoperative dressing, the tied vessel loop was pulled slightly and fixed again to keep the wound margin tight (Fig. 3B). On postoperative day 17, the wound was completely closed, and the patient was discharged without any adverse events.

An ultrasound taken at the 30-day postoperative follow-up showed that the basilic vein was ≥ 6 mm, and flow volume was ≥ 1000 ml/min, with the wound healed completely. From that day, hemodialysis was performed via the transposed basilic vein without any problems (Fig. 3C). Physical examination and duplex sonography performed at the 3-month, 6-month, and 12-month follow-ups revealed no problems, and the patient has been undergoing hemodialysis without any problems for 15 months since the surgery. Informed consent was obtained from the patient for the publication of the case report and the accompanying image.

## 3 Discussion

AVF aneurysms occur as a result of pre-stenotic or post-stenotic dilatation caused by excessive high flow or access stenosis, and most cases result from prolonged use of a fistula.^[4,9]^ Most reported cases of AVF aneurysms were limited to the forearm or upper arm. Reports of surgical treatment of a GVA that extensively spans the mid forearm to the upper arm, such as our case, are scarce. Because there is no established guideline for the timing of treatment for GVA, the risks and benefits should be weighed appropriately, and the most optimal surgical approach must be chosen.

The size of a venous aneurysm alone is not an indication for surgical treatment. Even with large aneurysms, therapeutic interventions are unnecessary if there are no problems with hemodialysis and the patient has no discomfort. However, even if there are no issues with hemodialysis, treatment is essential if the enormous size of the aneurysm causes pain or discomfort due to heaviness or the patient shows hemodynamic changes, such as steal syndrome or high-output cardiac failure. Furthermore, poor cosmetic appearance due to the GVA, as was true for our patient, is not negligible.

Although various treatment modalities have been introduced for symptomatic aneurysms, definitive treatment guidelines are lacking.^[10,11]^ Endovascular treatment, a noninvasive approach in which the aneurysm is excluded with a stent-graft, has been attempted.^[12,13]^ However, because this method hinders cannulation for hemodialysis in the stent-graft insertion site, despite maintaining the patency and function of the access itself, it should only be selectively considered in special cases, such as patients with a healthy section of vein for cannulation and patients with contraindications for surgery or patients without a surgical alternative.

In addition to aneurysm resection, aneurysmorrhaphy using plication, a stapling device, and an external prosthesis has been introduced as a surgical treatment for AVF aneurysms to reduce the volume of the aneurysm sac while maintaining the access by re-fashioning the aneurysm.^[7,14]^ However, these surgical treatments are not a viable option for GVA, where the aneurysm itself is enormously enlarged or has severe tortuosity and there is thrombus formation or wall thickening in the aneurysm sac. In addition, patients must continue undergoing cannulation, so inappropriate remodeling increases the risk for recurrence.

Although the surgical treatment for GVA does not markedly differ from that of AVF aneurysms, two things must be taken into consideration. First, a new dialysis access needs to be created after removing the aneurysm, and second, the large wound caused by the resection of the enormous aneurysm needs to be closed and healed.

In general, an artificial graft is interpositioned along with aneurysm resection to secure new dialysis access, but other alternatives need to be contemplated if the patient has an infection risk or does not want an artificial graft. In most cases of AVF, the cephalic vein is matured and is used as the cannulation site, while the basilic vein is preserved. Thus, the basilic vein should be examined preoperatively via ultrasound to ensure that it has an adequate diameter and length, and a surgical method utilizing a basilic vein segment, as opposed to an artificial graft, should be considered. In the present case, we confirmed via preoperative ultrasound that the patient had a good basilic vein, so we performed one-stage AVF formation during aneurysm resection by superficially positioning the exposed basilic vein and anastomosing it to the brachial artery. Particularly, patients who undergo surgery for an infection-related complication of aneurysm have a high postoperative infection risk, so such surgical approaches must be considered.

GVA causes thinning of the overlying skin as the aneurysm enlarges, and continuous cannulation also causes skin thinning. Hence, the skin defect remaining after aneurysm resection is too large for effective closure. To address this issue, we applied the vessel loop shoelace technique. This is a wound closure technique introduced to promote wound healing following fasciotomy in compartment syndrome, where the skin edge is gradually approximated in cases in which primary closure of the skin is difficult due to retraction of skin edge.^[15–17]^ Skin staples are placed alternately at the skin edge, and a vessel loop is run through the skin staples to maintain continuous traction. Then, the loop is tightened at 48-hour intervals to approximate the skin margin. One benefit of this technique is that it facilitates wound closure without inflicting excessive tension by maintaining continuous, mild traction based on the elasticity of the vessel loop.

When the vein is excessively dilated by a GVA, a large defect remains on the skin and subcutaneous tissue after removing the venous aneurysm. In particular, the skin overlying the AVF is thinned because of chronic cannulation for hemodialysis, so in some cases it must be removed when designing the skin margin for closure following aneurysm resection. In such cases, a large wound occurs, and primary wound closure is difficult. However, with the vessel loop shoelace technique, the areas in which the skin can be pulled are approximated through skin traction as much as possible, and a vessel loop is run around the remaining areas for further traction every 2 to 3 days to facilitate the approximation of the skin margins, such that the wound can be closed without additional procedures such as skin grafts and flaps.

## 4 Conclusion

Since GVA diminishes the quality of life and chances of survival of patients on hemodialysis, surgical treatment should be considered for symptomatic GVA. Concomitant aneurysm resection with AVF formation using the basilic vein followed by gradual closure of the large wound using the vessel loop shoelace technique is a safe and effective surgical procedure for GVA.

## Acknowledgments

The author would like to thanks Editage (www.editage.co.kr) for English language editing.

## Author contributions

**Data curation:** Young Sun Yoo.

**Investigation:** Young Sun Yoo.

**Methodology:** Young Sun Yoo.

**Visualization:** Young Sun Yoo.

**Writing – original draft:** Young Sun Yoo.

**Writing – review & editing:** Young Sun Yoo.
